# Peripheral blood RNAseq links neutrophilic inflammation to clinical glioma metastasis

**DOI:** 10.1186/s12916-021-02174-3

**Published:** 2021-12-03

**Authors:** Yuanyuan Wu, Zhichao Fan

**Affiliations:** Department of Immunology, School of Medicine, UConn Health, 263 Farmington Ave, Farmington, Connecticut 06030 USA

**Keywords:** Gliomas, Circulating tumor cells, Neutrophils, Neutrophil extracellular traps

## Background

Glioma is the most common intracranial tumor, representing about 60% of all brain tumors, and show varying degrees of malignancy [1]. Gliomas have a poor prognosis and can cause markedly high mortality. Tragically, glioblastoma (GBM), the most common glioma disease, reduces 5-year relative survival rate to ∼5% [2]. Thus, the mechanistic study of gliomas has vital clinical significance.

## CTCs and CTC clusters

Liquid biopsies from cancer patients can enhance prognosis prediction and treatment accuracy. Many risk factors have been examined as potential contributors to glioma risk, and these studies offer new insights into the diagnostic and therapeutic strategies to suppress cancer metastasis. Analysis of circulating tumor cells (CTCs), which are highly efficient metastatic precursors, is reported to be a promising method to study the mechanisms of tumor cell dissemination and metastasis formation [3, 4]. CTCs can be found in the blood of cancer patients as single cells or as CTC clusters. While research on glioma CTCs has been slow compared with other tumor types, more attention has been paid recently. First shown to be present in GBM in 2018, CTC clusters ranging from 2 to 23 cells were present at multiple sampling time points in a GBM patient with pleomorphism and extensive necrosis throughout disease progression [5].

## hTERT helps identifying postoperative CTCs as a poor prognosis factor

The paper presented by Zhang et al. [6] provides a more sensitive CTC measurement than the conventional CellSearch method. Their method, which is based on human telomerase reverse transcriptase (hTERT) detection, revealed new mechanisms between CTCs and innate immunity, especially pathways of neutrophil activation and neutrophil extracellular traps in gliomas. The hTRET detection method is a cell-surface marker-independent technology. It uses telomerase-specific, replication-selective oncolytic herpes simplex virus-1 to target telomerase reverse transcriptase-positive cancer cells and label them with green fluorescent protein, enabling identification of viable CTCs from a broad spectrum of malignancies [7]. Using this method, Zhang et al. found that postoperative CTCs are a poor prognosis factor, while preoperative CTCs are not, which refines our former understanding of glioma CTCs [6].

## RNAseq links postoperative CTCs with neutrophil inflammation

Understanding mechanisms of metastasis not only involves CTCs but also their interactions with circulating immune cells. Another recent study used single-cell RNAseq to find that cooperation between neutrophils in the immune system and CTCs in the blood of patients with breast cancer can promote CTC proliferation and metastasis [8]. According to this new mechanism, the innate immune system may cooperate to drive tumor progression [9]. By analyzing RNAseq data of peripheral leukocytes in glioma patients, Zhang et al. further demonstrated that postoperative CTCs might stimulate peripheral innate immune responses by activating neutrophils and generating neutrophil extracellular traps (NETs), exhibiting increased cell cycle and DNA replication programs [6]. On the other hand, postoperative CTCs were negatively correlated with the cytotoxic response [6]. This is the first study on the correlation between macro-immunity and CTCs in glioma patients. This finding may provide new ideas for targeted systemic immunomodulatory therapy in patients with gliomas and an opportunity to reduce their spread.

## Conclusions

While these studies have established the high diagnostic value of glioma CTCs, the precise detection of glioma CTCs is challenging because of physiological features such as circulation dynamics and the lack of consistently expressed tumor markers [10]. Besides, CTCs remain a limited exhibition of the metastatic process due to their dilution in patients’ blood and the challenge to isolate them [5]. Therefore, omnipresent and specific markers of glioma CTCs in blood should be pursued in future research. Although neutrophil-mediated inflammatory responses correlate with postoperative CTCs and prognosis in GBM, more mechanistic studies are needed to identify potential molecular targets that reveal new insights for GBM treatment.

## Data Availability

Not applicable.
